# Room-Temperature Plasticity of a Nanosized GaN Crystal

**DOI:** 10.1021/acs.nanolett.1c00773

**Published:** 2021-07-27

**Authors:** Masaki Fujikane, Shijo Nagao, Dariusz Chrobak, Toshiya Yokogawa, Roman Nowak

**Affiliations:** † Applied Materials Technology Center, Technology Division, Panasonic Corporation, 3-4 Hikaridai, Seika-cho, Soraku-gun, Kyoto 619-0237, Japan; ‡ Institute of Scientific and Industrial Research, Osaka University, Osaka 567-0047, Japan; § Extreme Energy-Density Research Institute, Nagaoka University of Technology, Nagaoka, Niigata 940-2188, Japan; || Opto-Energy Research Center, Depatment of Materials Science & Engineering, Yamaguchi University, Yamaguchi 755-8611, Japan; ⊥ Nordic Hysitron Laboratory, School of Chemical Engineering, Aalto University, Aalto 00076, Finland

**Keywords:** GaN nanocrystals, nanoscale compression, plasticity, ultrahigh voltage electron microscopy, *ab initio* calculations, MD-simulations

## Abstract

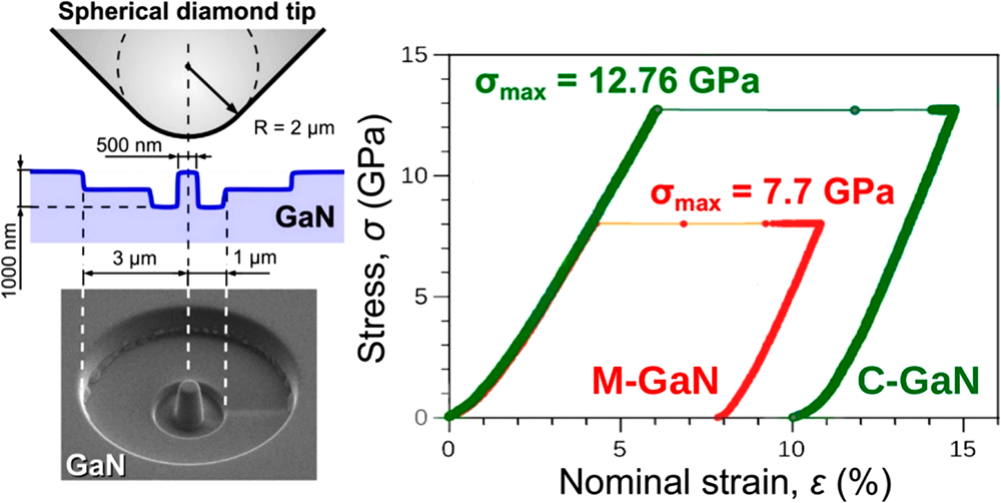

GaN wurtzite crystal is commonly regarded as eminently brittle. However, our research demonstrates that nanodeconfined GaN compressed along the M direction begins to exhibit room-temperature plasticity, yielding a dislocation-free structure despite the occurrence of considerable, irreversible deformation. Our interest in M-oriented, strained GaN nanoobjects was sparked by the results of first-principles bandgap calculations, whereas subsequent nanomechanical tests and ultrahigh-voltage (1250 kV) transmission electron microscopy observations confirmed the authenticity of the phenomenon. Moreover, identical experiments along the C direction produced only a quasi-brittle response. Precisely how this happens is demonstrated by molecular dynamics simulations of the deformation of the C- and M-oriented GaN frustum, which mirror our nanopillar crystals.

Semiconductors have been the life blood of technology ever since the first contact-point transistor was invented in 1947.^1^ It would seem therefore that every aspect of semiconducting materials has been thoroughly investigated,^2^ yet their mechanical properties have received significantly less attention than the optoelectronic ones. The majority of inorganic semiconductors are stiff, barely deformable solids with powerful interatomic bonding reflected by high melting temperatures *T*_m_.^3^ However, more recent research has challenged that idea with the synthesis of a first ever plastic Ag_2_S inorganic semiconductor^4^ or the discovery of plasticity of ZnS crystals under complete darkness,^5^ whereas the high-temperature mechanical behavior of ceramic and semiconductor nano-objects has been attracting increasing attention of the scientific world.^6^ Our own findings show that even a brittle semiconductor such as GaN can, under appropriate conditions (a nanodeconfined state^7,8^ and straining in a particular direction), display distinct plasticity even at room temperature (RT). At the same time, Kamimura, Kirchner, and Suzuki^9^ have estimated that GaN’s transition from brittle to ductile would require a temperature of 800 °C, suggesting that any talk of GaN’s plastic behavior would be possible only in the context of high temperatures. It is little wonder then that GaN’s plastic deformation at room temperature is utterly expected. This is also a reason why GaN plasticity has previously been methodically examined exclusively in high temperatures.^10,11^

GaN crystal has a well-established place in modern technology.^12,13^ In bulk mode and room temperature, GaN has long been known to display considerable strength, hardness, and brittleness.^14−17^ The same applies to GaN nano-objects that Huang et al.^18^ characterized as displaying only limited local plasticity in contrast to the “global” one exhibited by metallic micro-objects (cf. studies by Nix,^19^ Minor,^20^ or Schuh^21^ and their research teams). Despite wide-ranging testing conditions,^18,22−26^ no such “global GaN plastic behavior” has been detected, yet our Letter proposes that it is real. We turn therefore to GaN crystal, whose structure—unlike that of other semiconductors, e.g., GaAs^27,28^ or Si^29^—has been shown to remain stable until the pressure reaches 47–60 GPa.^30−33^

We began by performing density functional theory (DFT) calculations of the bandgap *E*_g_ in a stressed GaN structure (Supporting Information A-1), using the *Quantum Espresso* software package.^34,35^ The exchange-correlation energy was determined according to the Perdew–Burke–Ernzerhof functional.^36^ The ultrasoft Ga and N pseudopotentials were selected from the *PSLibrary* database.^37^ The energy cutoff of 60 Ry was established as the threshold for the wave function expansion, whereas the first Brillouin zone was sampled by applying the 11 × 11 × 11 Monkhorst–Pack *k*-point mesh^38^ (Supporting Information A-1 and Figure S1).

The obtained quasi-linear rise of *E*_g_ with increasing hydrostatic pressure *p* (Figure 1) agreed with earlier experimental observations,^39−42^ lending credibility to our calculations (see Supporting Information B). However, the declining *E*_g_–ε relationship found for GaN when compressed along the M[101̅0] direction (marked in red) was quite unexpected, diverging both from the hydrostatic (marked in black) and the C[0001]-axis stressing (marked in green). It implies a stark contrast in GaN’s mechanical response depending on whether it is stressed along the M- or C-axis (Figure 1), where earlier reports^13,30,43^ have considered its elastic anisotropy as insignificant.

**Figure 1:**
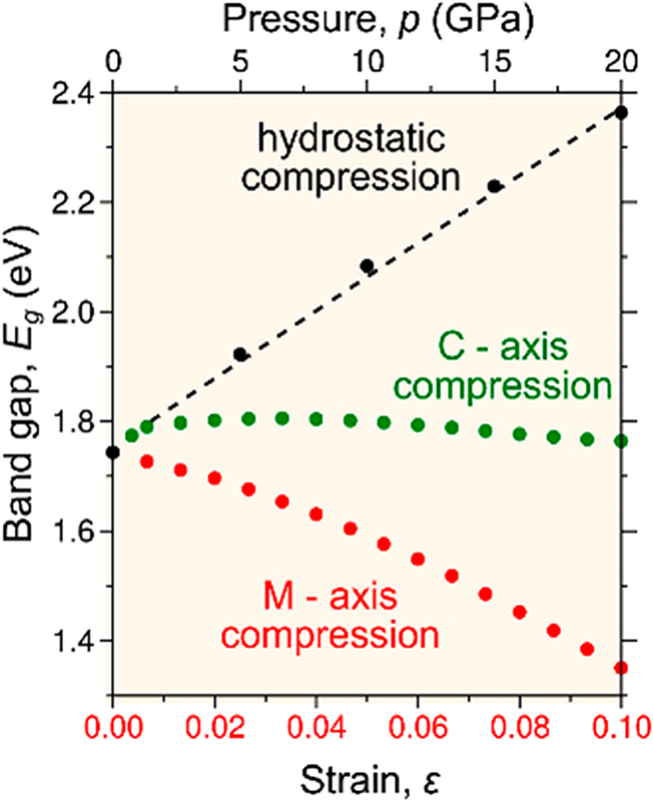
Stress-dependent changes of GaN bandgap deduced from ab initio calculations. The black dotted line (the upper pressure scale) concerns hydrostatic compression of GaN structure; the red and green data relate to the crystal compression (red scale refers to uniaxial strain) along the M[101̅0] and C[0001] directions, respectively.

Topical experiments by Porowski et al.^44^ show the GaN bandgap commensurate with its melting temperature, and an increasing *T*_m_*–p* dependence. Because the *T*_m_ value reflects crystal cohesion (Supporting Information B), it is reasonable to expect crystal “weakening” during straining along the M direction (Figure 1). This significant loss of strength in GaN persuaded us to undertake an experimental assessment of its nanoscale deformation along the M direction, which, to the best of our knowledge, had never been attempted before.

Consequently, GaN wafers (5 × 5 × 0.4 mm) with the C(0001) and M[101̅0] oriented surfaces were cut from a larger crystal grown by hydride vapor phase epitaxy in a way to avoid defect generation. An initial cathode luminescence examination of the materials confirmed their high quality: the threading dislocation density in C- and M-oriented wafers equaled 1 ×10^10^ and 4 × 10^9^ m^–2^, respectively. A set of virtually identical GaN nanopillars was carved in each of the prepared wafers using a two-stage focused ion beam (FIB) milling (Supporting Information A-2 and Figure S2), taking every precaution the FIB did not introduce dislocations in the fabricated items.

The RT nanocompression tests followed the approach developed by Schuh and his team^21^ as well as the updated standards for GaN nano-objects reviewed by Fatahilah et al.^45^ The experiments were carried out using a nanoindenter with precise test-geometry (Figure 2a), by compressing each pillar to a different force-limit in an effort to elicit a single strain-burst response (Supporting Information A-3 and Figure S6). This strategy proved successful, revealing an entire, irreversible deformation of certain C-GaN and M-GaN nanopillars accomplished under constant nominal stress σ_max_ (Figure 2b). The recorded stress–strain curves display a disparity between the M-GaN and C-GaN nano-objects, with displacement bursts occurring at different stress levels, namely, σ_max_ = 7.7 (red curve) and 12.7 GPa (green curve), respectively. The latter conforms to the RT hardness of GaN thick-films of *H* ≈ 12 GPa,^13,14^ leading to the conclusion that the C-GaN case represents the commonly recognized mechanical properties of GaN crystal,^13−17,25,26^ whereas the M-GaN one corroborates the “unexpectedly weak behavior” foreseen by ab initio calculations (Figure 1). Our claim is strengthened further by an inspection of nanopillars deformed according to a single strain-excursion pattern using scanning electron microscopy (SEM), which exposed the difference between a brittle-ceramic manner of the C-GaN deformation (Figure 2c) and plastic performance by the M-GaN (Figure 2d). Credible proof of this idiosyncrasy came from ultrahigh-voltage (1250 kV) transmission electron microscopy (UHV-TEM), which enabled first-hand observation of GaN structure in our 500 nm thick nano-objects (Supporting Information C-1 and C-3 and Figure S7). The deformed C-GaN nanopillar contains a huge number of accumulated dislocations (Figure 2e) and a vertical crack, which confirms its quasi-brittle response. This is similar to the effect found for GaN nanowires by Huang et al.^18^ and the conventional view of GaN properties.^13−17^ Like the majority of earlier works on compressed GaN micropillars, they report a vertical crack, which is due to their exclusive concentration on C-oriented objects.^22−24^

**Figure 2:**
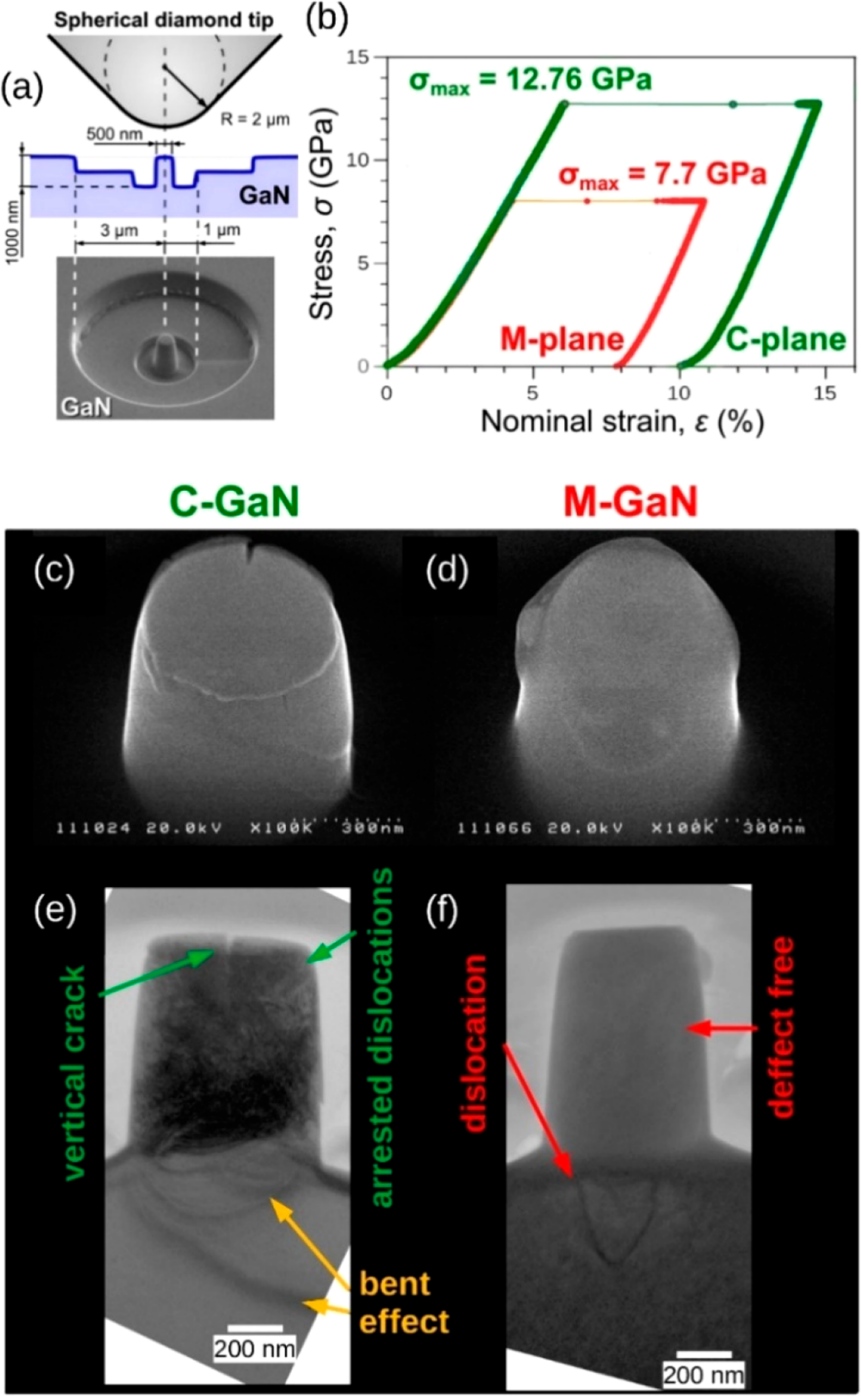
Results of nanocompression experiments carried out on the C- and M-oriented GaN nanopillars: (a) a schematic of the performed test, (b) the stress–strain σ–ϵ curves determined for the C-GaN and M-GaN nanopillars, the general SEM views of the postdeformed (c) C-GaN and (d) M-GaN crystals. Also included are bright-field see-though views of the entire (e) C-GaN and (f) M-GaN pillar structure that remains after the single strain-burst deformation. (e) The C-oriented GaN reveals a significant accumulation of stacked dislocations as against the M-GaN, which manifests a defect-free crystalline structure. The displayed quasi-brittle response of C-GaN with (c, e) a vertical crack formation contrasts with the plasticity evident in (d, f) the M-GaN. (See also the structure observed for inclined nanopillars in Figures S7 and S8.)

By contrast, our results concern a defect-free M-GaN structure obtained after severe (ε_M-GaN_ = 8%) irreversible deformation (Figure 2b). As it happened, thorough microscopic observations of the whole volume of the M-oriented pillars variously inclined to the incident electron beam (see Figure S8) failed to detect a single dislocation. There is no doubt that, if any such defects in the nano-object structure existed, they would have left a trace in bright-field UHV-TEM images (Figure 2f), similarly to the defects arrested within the C-GaN (Figure 2e). The absence of dislocations in the M-GaN pillar on the one hand, and their existence in the confined root-substrate (Figure 2f and Figure S8) on the other, is ample proof that dislocation activity did indeed occur, causing the hard GaN crystal to deform through slip. It bears emphasizing that the phenomenon we are dealing with differs from “dislocation starvation” or “mechanical annealing” reported for metallic nano-objects,^19,20,46^ concerning as it does a strong solid with considerable resistance to dislocations motion.^16^

We have tried to account for the experimental data using MD simulations. Our computations were performed with the LAMMPS code^47^ for two frustum (Φ_top_ × *H* × Φ_bottom_) objects: C-GaN (14.7 × 29.8 × 17.9 nm) and M-GaN (14.9 × 29.9 × 17.7 nm) placed on a GaN wafer, which reflect the geometry of the examined samples. The interactions among the atoms within the wurtzite structure were described using the Stillinger–Weber^48^ potential created by Béré and Serra,^49^ who demonstrated its accuracy in modeling GaN lattice parameters and elastic constants (refer to Supporting Information A-4). The deformation path of each object was induced at 300 K by applying a load to the rigid, horizontal plate while in contact with the upper frustum surface. To achieve a quasi-static deformation, we relaxed the plate shift increments of 0.3 Å within 2 ps time intervals (details in Supporting Information A-4).

The MD-simulated deformation history of C-GaN frustum displayed in the contact pressure-strain (*p*_c_–ε) curves (Figure 3) conforms qualitatively with the experimental data obtained for compressed GaN microprisms by Wheeler et al.,^24^ or those by Huang et al.^18^ for GaN nanowires squeezed along their C[0001] axis. This agrees with a common perception of GaN objects as stiff and brittle materials in a range of testing temperatures.^13,17^ However, the simulated *p*_c_(ε) characteristics unveiled a noticeable difference in the mechanical conduct of the C- and M-oriented frustums quite unlike the moderate directionality registered in our earlier nanoindentation experiments on bulk GaN crystal.^25,26^

**Figure 3:**
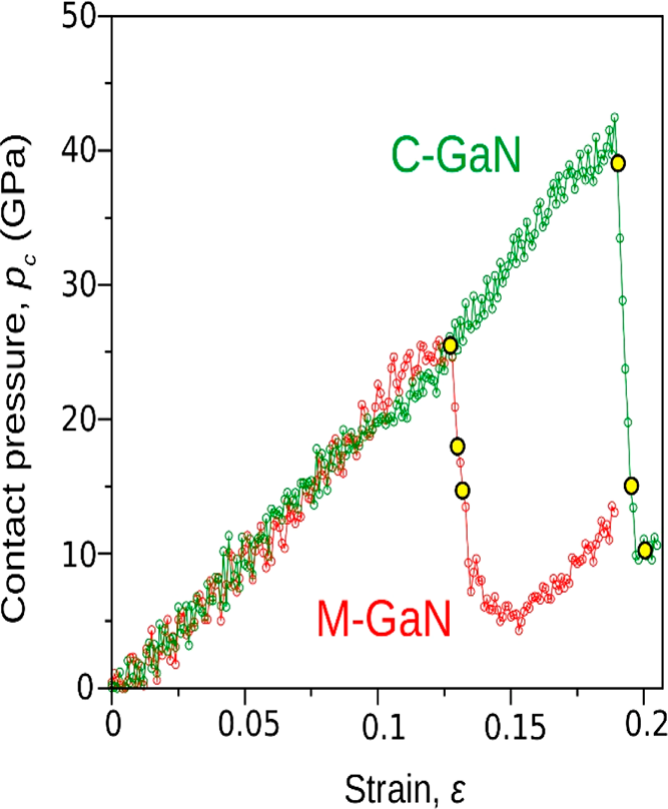
Contact pressure–strain *p*_c_–*ε* relationships for the C- and M-oriented GaN frustums compressed at 300 K show a difference in mechanical response of nano-objects. The abrupt pressure-drops in *p*_c_–*ε* graphs that correspond to a single-burst deformation (Figure 2b) confirm considerably stiffer behavior of the C-GaN, whereas the M-oriented frustum starts to deform under significantly lower stress. A visualization of structure evolution under increasing strain ε (selected stages of compression marked by yellow points) is presented in Figure 4.

The abrupt stress-drop recorded in both *p*_c_(ε) relationships (Figure 3) corresponds to the strain bursts (Figure 2b and Figure S6), as MD simulations commonly apply depth-controlled compression, whereas nanomechanical testing is load-controlled.^7,50^ It turns out that the C-GaN requires a significantly higher contact pressure than the M-GaN to initiate irreversible deformation (Figure 3), which accords with our experiments (Figure 2b). However, neither the simulated nor measured mechanical characteristics of C- and M-oriented nano-objects (Figure 2b and 3) appear to resolve the dilemma we are in, namely, of a sizable plastic M-GaN deformation with a resulting intact crystalline structure on the one hand, and a quasi-brittle response of the C-GaN with arrested dislocations (Figure 2) on the other.

The answer was provided by a visualization of the evolution of a strained atomic GaN structure recently made available with *OVITO* (Open Visualization Tool) and the dislocation extraction algorithm (DXA).^51^ In particular, our employment of the atomic shear strain modifier enabled us to envision the shear strains that atoms are subjected to and determine active slip planes, existing dislocations, and their Burgers vectors^52^ (see Supporting Information D-1). We found that the onset of an irreversible C-GaN deformation (Figure 3) concerns defect activity at the lower end of the modeled pillar (refer to Figure 4a). It involves limited local, quasi-elastic movement of dislocation lines on the C planes (DXA-details in Supporting Information D and Figure S3) that leads to accumulation of defects inside the crystal. The irreversible deformation starts with dislocation generation at the base of the frustum (Figure S3) despite the inevitable presence of higher stress close to its upper end (smaller cross-section area). We contend that the loading of a significantly strong and stiff C-GaN nanopillar results in a moderate stress concentration along the perimeter of the bottom contact and local change in the GaN lattice orientation.

**Figure 4:**
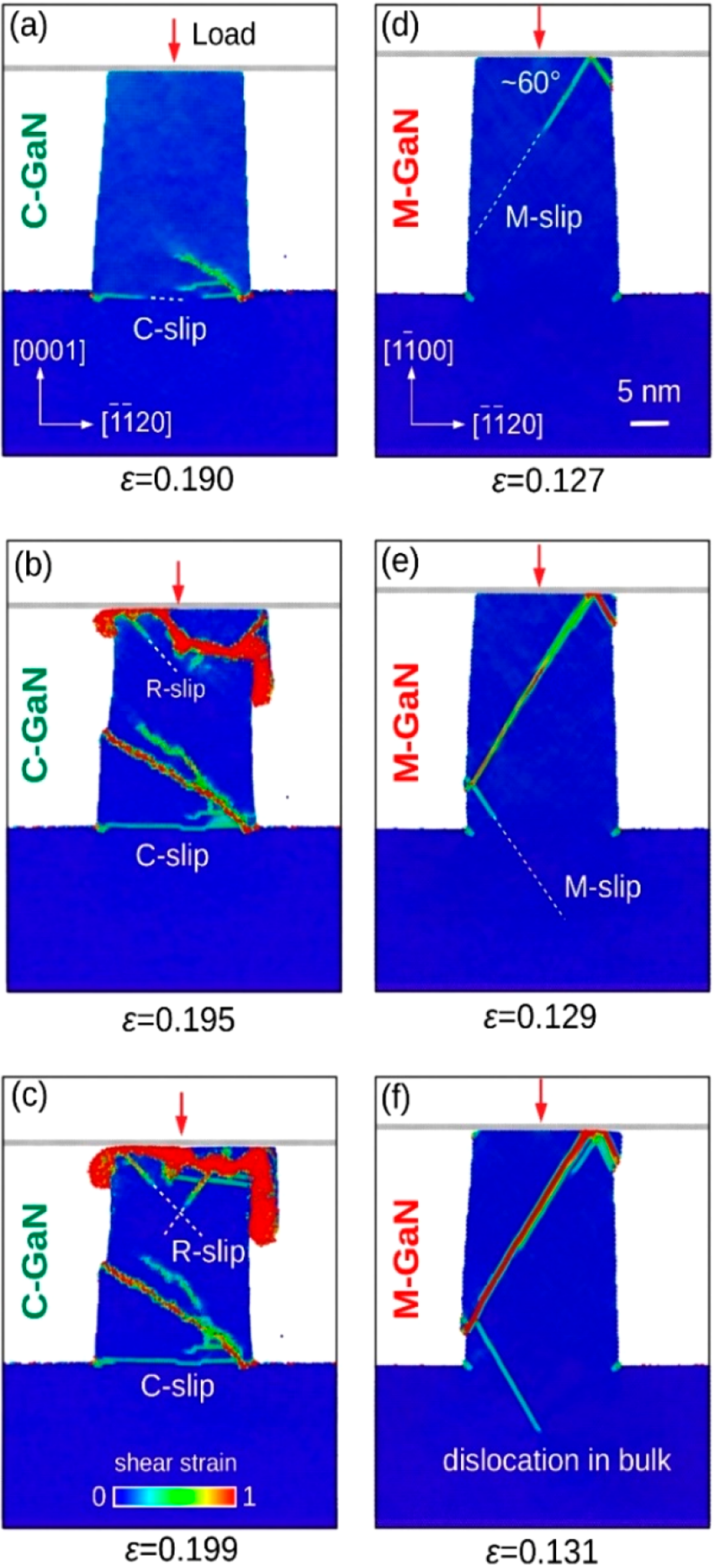
Contrasting structure evolution during compression of the (a–c) C- and (d–f) M-oriented GaN frustums derived from our MD simulations. The sequence of selected strain values complies with the yellow points in *p*_c_–*ε* curves for both objects (Figure 3), whereas the depicted structural changes are exposed in the vertical, diameter cross-section of a modeled pillar. The atoms are marked in colors according to the atomic shear stress level determined in their actual location. (a–c) Massive accumulation of defects in the C-GaN frustum and (d–f) loss of its integrity due to crystal-block sliding and the extrusion of stressed material are in marked contrast to the plasticity of the M-GaN realized by multiple M slips.

As deformation proceeds, the R slip operates in the upper part of the nano-object and forms a typical “mushroom profile” of extruded material close to the contact (Figures 4b, c), similarly to the observations by Huang et al.^18^ In reality, stress relaxation is realized by outward movement of the material immediately below the squeezing tool (with only a limited contribution from the C and R slip) and by vertical cracking of the C-GaN pillar which both we (Figure 2c, e) and other authors^22−24^ have observed. The lion’s share of the dislocations generated in the C-GaN frustum is stacked in the crystal volume, as demonstrated by our simulations (Figure 4a–c) and UHV-TEM observations (Figure 2e), because they are unable to escape the pillar volume or slip either during deformation or unloading (Supporting Information Figure S4).

The visualization highlights the plastic response of the M-GaN (Figures 4d–f and 3) in marked contrast to the vast defect accumulation in the compressed C-GaN (Figure 4a–c). This kind of C-GaN’s “quasi-brittle” behavior is consistent with the common perception of the mechanical properties of our nitride.^13−17,30,43^ Particularly revealing, however, is the unobstructed, well-defined, multiple dislocation slip on the M planes right across the M-GaN crystal (Figure 4d, e), with some of it entering the substrate area (Figure 4f), which our TEM experiments had detected (Figure 2f). Indeed, the Peierls–Nabarro stress for the GaN prismatic M⟨112̅0⟩{11̅00} slip, claimed by Kamimura et al.^53^ as well as Yonenaga and Motoki^54^ to be lower than the Peierls barrier for other slip systems, indicates the possibility of the M slip.

One final piece of the puzzle was missing: why should the above mechanism result in a dislocation-free M-GaN structure (Figure 2f) despite the sizable plastic deformation (Figure 2b)? Our MD simulations of the loading path (Figure 4) showed that, similarly to the C-GaN, the M-oriented frustum initially also contains dislocations, although to a much lesser degree (compare Figure 4d–f and Figure 4a–c). In search for the answer, we investigated the evolution of defects during the unloading of the M-GaN. The DXA revealed that the generated dislocations (Figure 4d–f) did not in fact contract during unloading (Figure 5). Instead, they expand and annihilate themselves on the lateral surface (Figure 5b, c, f, g), leaving behind a perfect GaN structure (details in Supporting Information D-2 and Figure S5), in full confirmation of our UHV-TEM observations (Figure 2f). Some other dislocations located close to the bottom of the frustum (Figure 5i, m) enter the substrate (Figure 5j–l and Figure 5n–p) during unloading, again in accordance with our experiments (Figure 2f). Taken together, both our experiments and simulations stipulate that a specifically oriented GaN nanopillar will not perform like a brittle material.

**Figure 5:**
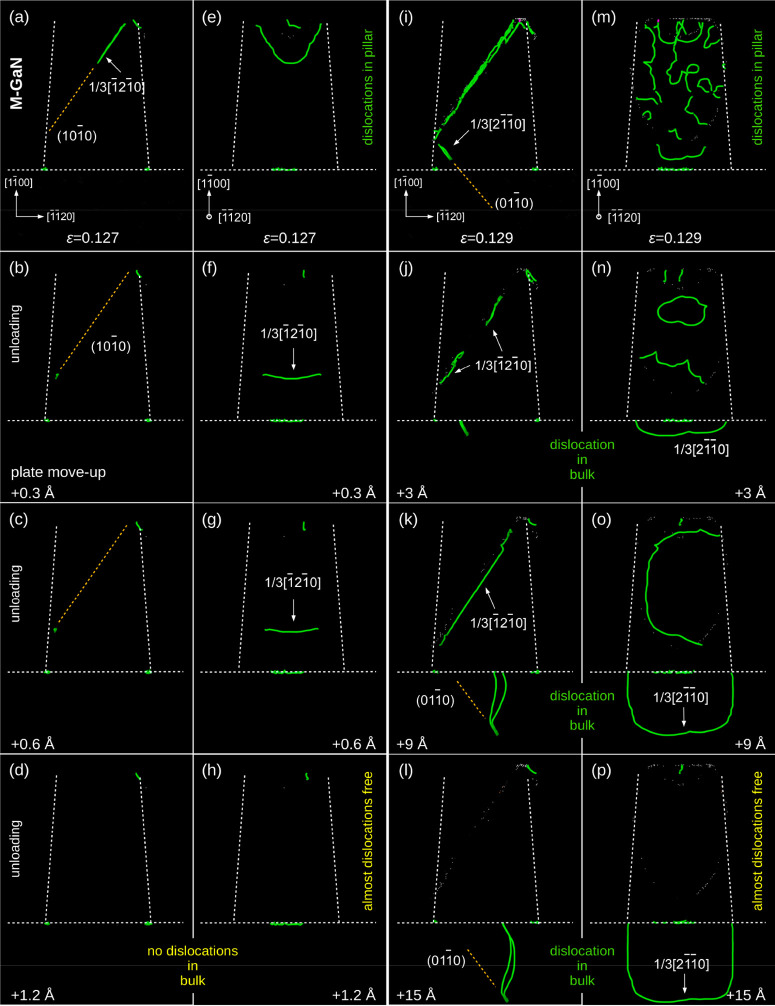
DXA visualization of dislocations in the M-GaN frustum (a, e) strained up to ε = 0.127 and (b–h) after unloading. Panels a and e concern the pillar viewed along two different, perpendicular directions. Similarly, in the M-GaN frustum (i, m) prestrained up to ε = 0.129 and (j–p) unloaded. (a–h) Significantly, the dislocation induced during loading disappears on reaching the lateral surface during unloading. However, in the larger prestrained frustum (i) and (m), (i–p) the relaxation process results in the annihilation of defects except for a single one (l and p) that extends into the M-oriented root substrate. The additional description of unloading stages for differently strained M-GaN is provided in Supporting Information D and Figure S5.

Contemporary developments in GaN fabrication^55^ are opening the way to appliances capable of outperforming Si-based products. They include nanocolumn LEDs,^13^ the next generation of power-electronic devices, or wirelessly powered systems in autonomous cars.^56,57^ Consequently, we are witnessing increased demand for all-embracing knowledge of the mechanical properties of GaN nanovolumes.^58^ The plastic response of M-oriented GaN nano-objects goes some way toward meeting that demand.

## Supporting Information Available

Details of the numerical and experimental methods; discussion on a stress-dependent bendgap-melting temperature *E*_g_–*T*_m_ relationship for GaN; additional experimental particulars; (D) MD-based analysis (Figures S3–S5) of GaN nanoscale deformation; (E) supplementary references; and Figures S1–S8 (PDF)

## Abbreviations

MD: Molecular Dynamics
UHV-TEM: ultrahigh voltage transmission microscopy
DXA: dislocation extraction algorithm
