# Supplementary material for: Room-Temperature Plasticity of a Nanosized GaN Crystal
Source: Nano Lett. 2021 Jul 27;21(15):6425–31. doi: 10.1021/acs.nanolett.1c00773 (PMC8397389; doi:10.1021/acs.nanolett.1c00773)
Supplement: Supplementary file 1 — nl1c00773_si_001.pdf [file nl1c00773_si_001.pdf]

Supporting Information:

Room-temperature Plasticity of a Nanosized GaN Crystal

Masaki Fujikane,[†,] Shijo Nagao,[‡] Dariusz Chrobak,[§,⊤] Toshiya Yokogawa,[‖] Roman Nowak [‡,§,⊥,*]*

[†] *Applied Materials Technology Center, Technology Division, Panasonic Corp., 3-4 Hikaridai, Seika-cho, Soraku-gun, Kyoto 619-0237, Japan*

[‡] *Institute of Scientific and Industrial Research, Osaka University, 567-0047 Osaka, Japan*

[§] *Extreme Energy-Density Research Institute, Nagaoka University of Technology, Niigata 940-2188, Japan*

[‖] *Opto-Energy Research Center, Dept. Materials Science & Engineering, Yamaguchi University, 755-8611 Japan*

[⊥] *Nordic Hysitron Laboratory, School of Chemical Engineering, Aalto University, 00076 Aalto, Finland*

Corresponding Authors

* (Masaki Fujikane – Panasonic Corp., Japan; Email: fujikane.masaki@jp.panasonic.com)

* (Roman Nowak – Aalto University, Finland; Email: roman.nowak@aalto.fi).

Contents:[1] page

A. A detailed description of the employed methods 2

A-1) *A first-principle evaluation of bandgap in stressed GaN* 2
A-2) *Fabrication of C- and M-oriented GaN nanopillars* 2
A-3) Nanomechanical experiments and structure observations 3
A-4) Molecular dynamics simulations 3

B. Bandgap and melting temperature of stressed GaN crystal 4

C. Additional experimental particulars 5

C-1) *Structure of GaN nanopillars* 5
C-2) *Nanoindentation of GaN wafers and compression of GaN nanopillars* 5
C-3) *UHV-TEM observations* 6

D. MD-based analysis of GaN's nanoscale deformation 7

D-1) *The slip responsible for plastic deformation of M-GaN* 7
D-2) *The defect-free structure of plastically deformed M-GaN nanoobjects* 7
D-3) *Plasticity of GaN nanocrystals in general context* 8

E. Supplementary references 9

F. Supplementary figures (1-8) 11

[1] The numbers in brackets [x] define the references in a main body of the Letter, while the supplementary references are denoted as [Sx].

A. A detailed description of the employed methods

A-1) *A first-principle evaluation of bandgap in stressed GaN:*

The *ab initio* DFT-calculations of the bandgap energy E_g in a compressed GaN structure were accomplished using the Quantum Espresso software package, whose formalism relies on a plane-wave expansion of electronic wave functions [34,35]. The exchange-correlation energy was determined according to the Perdew-Burke-Ernzerhof functional [36]. The ultrasoft Ga and N pseudopotentials were selected from the PSLibrary database [37]. The energy cutoff of 60 Ry was established as the threshold for the wave function expansion, while the first Brillouin zone was sampled by applying the 11×11×11 Monkhorst-Pack *k*-point mesh [38]. As a result, we were able to compress the wurtzite GaN lattice, defined by the $\boldsymbol{a}_H$, $\boldsymbol{b}_H$ and $\boldsymbol{c}_H$ vectors (refer to Fig. S1) both hydrostatically and uniaxially, along the C[0001] direction, while monitoring the variations in the lattice parameters and bandgap E_g. On the other hand, examining a unit cell squeezed along the M[$10\bar{1}0$] direction required reference to the orthogonal version of the GaN lattice, which involves: $\boldsymbol{a}_O=\boldsymbol{a}_H$, $\boldsymbol{b}_O=2\boldsymbol{b}_H+\boldsymbol{a}_H$, $\boldsymbol{c}_O=\boldsymbol{c}_H$ (Fig. S1)

A-2) *Fabrication of C- and M-oriented GaN nanopillars:*

GaN wafers (size of 5×5×0.4 mm) with the C(0001) and M($10\bar{1}0$) oriented surfaces were cut from a larger crystal, grown by Hydride Vapor Phase Epitaxy, in a way as to avoid defect-generation. An initial cathode luminescence examination of the materials confirmed their perfection, *i.e.*, the threading dislocation density in C- and M-oriented wafers equaled 1×10^{10} and 4×10^{9} m^{-2}, respectively. To fabricate proper specimens for nanomechanical testing, a set of identical GaN nanopillars was cut in each of the prepared wafers using a two-stage FIB-milling process (Fig. S2 and Fig. 2a). For clarity, the output of the first stage of cutting is shown in Fig. S2a, while the final result obtained after the second stage of the employed ion milling is displayed in the main text (Fig. 2a). The conditions of the initial stage of the employed FIB-etching are defined by the beam current level of 300 pA with its inclination (pitch) of 0.06. The process was accomplished in 800 shoots with a total duration of 81s. The second stage of fabrication was carried out with a current of 100 pA and pitch of 0.03 accomplished with 200 shoots within 12s.

The two-stage FIB-fabrication allowed us to use a large spherical tip to accomplish nanocompression tests without fear of the surrounding material coming in touch with the indenter. Furthermore, the fabrication of wide ‘craters’ were helpful in placing the tip exactly along the axis of each nanopillar. A final microscopic inspection of the obtained C- and M-oriented nanopillars confirmed their virtually identical shape and dimensions, along with a defect-free structure unaffected by the FIB-cutting. We deliberately produced fairly large nanopillars (500 x 1000 nm) in order to avoid buckling.

A-3) *Nanomechanical experiments and structural observations:*

Nanoindentation probing of C- and M-oriented wafers using a sharp and spherical tip provided us with fresh evidence of the insignificance of anisotropy in GaN's elastic and plastic behavior when in bulk state. This differs markedly from the response by deformed C-GaN and M-GaN nanoobjects.

The RT nanocompression experiments were carried out for the C-GaN and M-GaN nanopillars using the Hysitron TriboIndenter (TI-950) with the precise test-geometry defined in Fig. 2a. We compressed each nanopillar under a sizable spherical (nominal radius of R=2 μm) diamond tip loaded up to different peak-load values P_{max} ranging from 1 to 4 mN for C-GaN, and from 1.5 to 5 mN for the M-GaN nanoobjects. The duration of loading, dwelling and unloading was set arbitrary at 5s. The compression experiments with C- and M-oriented nanopillars were performed with a loading rate dP/dt varying from 0.2 to 0.8 mN/s and from 0.3 to 1.0 mN/s, respectively. The idea was to squeeze the nanopillars between rigid surfaces to deform the material under virtually uniaxial stress and to secure a single pop-in response. With the help of recorded load-displacement data, the stress-strain (σ–ε) relationship for a single-pop-in behavior was determined using the earlier developed procedure [7]. The structure of the severely deformed nanopillars was then subjected to SEM (Hitachi S5000) observations that and UHV-TEM (JEM-1300NEF@Kyusyu Univ.) which corroborated M-GaN's ductile behavior.

A-4) *Molecular dynamics simulations:*

In order to account for the experimental data we employed MD-simulations performed with the LAMMPS code [47]. The computations were carried out for two frustum (Φ_{top} x H x Φ_{bottom}) objects: C-GaN (14.7 x 29.8 x 17.9 nm,) and M-GaN (14.9 x 29.9 x 17.7 nm)] placed on a GaN wafer, that in total contained 3120538 and 3103745 atoms, respectfully. The employed clusters fairly reflect the geometry of the examined samples (refer to Fig. S2 and Fig. 2a). The interactions among the atoms within the wurtzite GaN structure were described by means of the Stillinger-Weber (SW) potential [48].

Our choice of the SW-potential was dictated by preliminary research and a survey of published information concerning different potentials, including the commonly used Stillinger–Weber, EDIP [S1], and Tersoff [S2]. A thorough analysis by Godet *et al*. [S3] suggested that SW was better suited to nanoobjects, securing as it does a close match with the *ab-initio* data for large shear strains and offering the best choice for modelling dislocation nucleation pertinent to the subject of our study.

Furthermore, a standard velocity-Verlet time integration with the time-increment of 1 fs was used through the simulations, while a Nose-Hoover thermostat and barostat were employed to control the thermo-dynamical variables. Prior to running compression simulations, each cluster was relaxed to the 'zero-force configuration', with thermal equilibration at the target temperature (300K). The deformation path of each cluster was induced by applying a load to the rigid, horizontal plate while in contact with the upper pillar surface. To achieve a quasi-static deformation, the plate shift increments of 0.3 Å were followed by the 2 ps structure relaxation selected as a time-interval for the system equilibration.

The recently available OVITO-software (Open Visualization Tool [51]) enabled us to precisely monitor the structure evolution of C- and M-strained GaN clusters, while the existing dislocations and their Burgers vectors were identified using DXA (dislocation extraction algorithm) modifier [52]. In particular, our employment of the "atomic shear strain" modifier within OVITO enabled us to picture the shear strains that atoms are subjected to, and to determine active slip planes.

B. Bandgap and melting temperature of stressed GaN crystal

Recent experiments carried out under a wide range of pressures (up to 10 GPa) revealed a systematic stress dependence of GaN melting point [44] in accord with earlier observations restricted only to narrow pressure intervals [40]. Furthermore, the bandgap energy of inorganic semiconductors scales with their melting point (T_m) [41,42], allowing an estimation of the crystal's bonding energy [42], *i.e.*, its strength. The changes of GaN lattice parameters under C- and M-uniaxial compression as well as hydrostatic deformation are displayed in Fig. S1.

Based on recent theoretical considerations of the correlation between bandgap, melting point, elastic modulus and size of crystalline GaN by Lu and Meng [S4], we begin our estimation with the electrical conductivity μ of crystals compressed in M and C direction:

$$\mu_M(\varepsilon, T) = \mu_0(\varepsilon)\exp\left(-\frac{E_{a,M}(\varepsilon)}{k_B T}\right), \qquad \mu_C(\varepsilon, T) = \mu_0(\varepsilon)\exp(-\frac{E_{a,C}(\varepsilon)}{k_B T}),$$

where E_C defines the edge of the conducting band, E_F is Fermi energy, while E_a energy reads: $E_a = E_C\text{-}E_F$. The electrical conductivity μ_C and μ_M would differ in principle at given temperature T and strain level of ε due to discrepancy in the bandgap energy (Fig. 1). Following the assumption by Lu and Meng [S4], we agree that one should expect $\mu_M = \mu_C$, at temperatures close to the melting point ($T \cong T_m$), which demands $T_{m,C} \neq T_{m,\mathrm{M}}$, since the bandgap energies are not the same in a strained crystal ($E_{g,M} \neq E_{g,C}$). This brings us to the relationship:

$$\exp\left(-\frac{E_{a,M}(\varepsilon)}{k_B T_{m,M}(\varepsilon)}\right) = \exp\left(-\frac{E_{a,C}(\varepsilon)}{k_B T_{m,C}(\varepsilon)}\right),$$

which yields:

$$\frac{E_{a,M}(\varepsilon)}{T_{m,M}(\varepsilon)} = \frac{E_{a,C}(\varepsilon)}{T_{m,C}(\varepsilon)}.$$

Using the basic relationship for semiconductors $E_a \cong E_g/2$ (see *e.g.*, Ref. S5) we arrive to the essential equation:

$$\frac{E_{g,M}(\varepsilon)}{E_{g,C}(\varepsilon)} = \frac{T_{m,M}(\varepsilon)}{T_{m,C}(\varepsilon)}$$

that confirms our prediction based on the available data that the bandgap energy scales with the actual melting temperature of a strained semiconductor. Consequently, the bond strength and cohesion of GaN crystal compressed in M-direction are lower than those of a one stressed along the C(0001) axis (Fig. 1). The revealed strong anisotropic behavior runs counter the common knowledge of GaN as displaying only moderate elastic anisotropy in contrast to the wurtzite AlN (see Ref. S6).

C. Additional experimental particulars

C-1) *Structure of GaN nanopillars*

As far as experimental data is concerned, we have reason to suspect that the quality of the crystals we have used (after ion-milling) is superior to that of the specimens used in previous research [23,24]. An important indicator is the C-GaN yield strength of 12.7 GPa (the highest among those reported for GaN nanoobjects) measured for our crystals (Fig. 2b), confirming a virtual absence of defects in our material. Frequent discussions with GaN producers have also convinced us that our free-standing nanoobjects (devoid of any interface) have a lower defect concentration than the epitaxial layers used frequently for pillars fabrication (these suffer from threading dislocations). Furthermore, we had performed UHV-TEM microscopic bright field observations of GaN nanopillar structure prior to compression, which appears defect free. It is impossible to compare the quality of our M-GaN with the crystals made by other researchers, as there is no experimental data for the M-oriented GaN except those we are presenting.

The relation between the mode of fabrication of nanopillars and their mechanical behavior is well known in nanoscience. However, it has been established that FIB is an efficient and adequate way of preparing nanopillars [S7]. Chen *et al*. who found the difference between the mechanical responses of GaAs nanoobjects prepared either by means of FIB or lithography to be far from essential [S7]. Moreover, the nucleation, movement and annihilation of defects during mechanical testing were not affected by radiation of any kind. The tester chamber was constructed in such a way as to act as the Faraday cage.

C-2) *Nanoindentation of GaN wafers and compression of GaN nanopillars*

In order to compare the mechanical behavior of GaN in its bulk (confined) and nanoobject (deconfined) state, we examined differently oriented (C and M) wafers as well as fabricated nanopillars. The nanoindentation data revealed negligible elastic and moderate plastic anisotropy, whereas the discrepancy between the mechanical response of the C-GaN and M-GaN nanoobjects appeared substantial (Fig. 2). A slightly stiffer elastic response of the C(0001) plane and more exposed plasticity of the M($10\bar{1}0$) plane – all in line with the simulations derived trend - did not match the remarkable effect stipulated by our microscopic observations and atomistic calculations. Our earlier experience with nanoindentation probing of GaN proved extremely helpful in these circumstances [15,25,26,S8-S13].

C-3) ***UHV-TEM observations***

We found no indication in the literature [S14-S17] that electron microscopy-induced irradiation of nanopillars would be capable of causing as massive glide as that anticipated for the M-GaN. Both the M-GaN and C-GaN nanopillars had been exposed to analogous irradiation conditions, causing dislocation movement of potentially comparable proportions. At the same time, there is no discernible defect healing in the C-GaN, which, unlike the M-GaN, remains defected after TEM observations.

Our research approaches dislocations and plasticity from a different angle, determining the selection of appropriate experiments. To begin with, it included a string of pillars with an aspect ratio of 1:2 to avoid buckling or virtual 'local plasticity' similar to that reported by Huang *et al*. [8] for GaN nanowires. Structural observations of nanoobjects reaching as much as 500 nm in diameter required the employment of the ultra-high voltage (1250 kV) transmission electron microscope, JEM-1300NEF, of the Ultra-microscopy Research Centre at Kyushu University. This enabled the investigation of the entire volume of the deformed nanopillar, thus rendering the complicated preparation of thin-film samples – which inevitably induces or anneals defects in the observed material - unnecessary.

The observations involved the C- and M-oriented nanopillars, whose inclination varied in relation to the incident electron beam (Fig. S7 and Fig. S8). Our examinations concerned bright field conditions in contrast to the dark-field investigations previously reported for compressed pillars [4-7]. It must be understood that our focus was on 'single strain-bursts' (Fig. 2b and Fig. S6), which we arrived at through trial and error, in order to extract the dominant mechanism of deformation.

The pillars were placed under the UHV-TEM in a number of angular positions (a tilt increment of $\Delta\alpha$=10°) with respect to the incident electron beam. Regardless of the applied tilt angle, our TEM observations were consistent in reviling the tangle of defects in the C-oriented nanopillar and their complete absence in the adjacent GaN base-substrate. (Fig. 2e and Fig. S7) The structure of the C-GaN nanopillar observed from different inclination angles: a=10° (a)(b) and a=0° (c,d) is shown in Fig. S7. The energy filtered method (EF-TEM, ΔE = 60 eV) was applied to obtain an improved picture quality of the severely deformed C-GaN structure (Fig. S7-b,d).

The same UHV-TEM procedure applied to the M-oriented GaN pillar revealed a contrasting post-deformation structure that consisted of dislocation-free pillar and the root-substrate with a sporadic dislocation in it (Fig. S8). Again, the inclination of the GaN-specimen to the incident electron beam had no bearing on the result: there was no trace of any dislocation in the M-GaN pillar (Fig. S8).

The output of our MD-simulations agrees with the experimental observations. The calculations indicated the local plasticity and a defected structure for the C-GaN (Fig. 2e and Fig. S7), whereas the M-GaN was characterized by the M-glide and perfect structure (Fig. 2f and Fig. S8).

D. MD-based analysis of GaN's nanoscale deformation

D-1) *The slip responsible for plastic deformation of M-GaN*

The identification of the slip system activated in GaN nanoobjects compressed along the M-direction, considerably relied on MD-simulations. As we demonstrated in the Letter, the M-GaN undergoes marked irreversible deformation (Fig. 2b), leaving behind – similarly to the MD-revealed scenario - a crystalline structure with no detectable defects (Fig. 2f). Under such circumstances, identification of activated slip-systems by means of TEM is a virtual impossibility.

However, we succeeded in identifying the pertinent M-slip system by resorting to the visualization of the MD-simulated, stressed GaN structure and DXA procedure, which enabled us to identify the active dislocation mechanisms and their development. These confirm that plasticity* of the M-oriented cluster is achieved by intense, multiple slip of dislocations on the selected M-$\{1\bar{1}00\}$ planes [Fig. 4(d-f)].

The heterogeneous mechanism of incipient plasticity, previously revealed by TEM examination of nanoindentation probed bulk GaN crystal [25,26,S10,S11], also applies to C-GaN nanopillars. It involves the formation of a metastable R-plane twin-boundary and subsequent activity of the R-slip, as reported by Fujikane *et al*. [26]. In the C-oriented GaN cluster, plastic flow starts with a limited, basal $\langle 11\bar{2}0\rangle(0001)$ slip (see Fig. S3) followed by slip in the R$\{1\bar{1}02\}$-planes [Fig. 4(b)], which taken together, contribute to a significant accumulation of the defects in nanocrystals [see also Fig. 2c, Fig. 2e and Fig. 4(a-c)]. Further, we performed additional MD-simulations with the Tersoff type potential by Nord *et al*. [S18]. The obtained results confirmed earlier calculations for an M-oriented GaN cluster using the Stillinger-Weber potential.

We believe that identifying the slip system by means of computational MD-simulations does not undermine the significance of our findings. Moreover, we have proved before [25,26,S10,S11] that incipient plasticity, viewed in terms of the first pop-in pressure, is independent of the C- and M-orientation for the bulk GaN crystal probed by nanoindentation. This is in stark contrast with the behavior of GaN nanoobjects, and enhances our understanding of the nanoscale deconfinement effect [7,8]. The issue is well in line with the recently growing interest in nanoscale deformation of important ceramic materials such as ZrO_2 [6].

D-2) *The defect-free structure of plastically deformed M-GaN nanoobjects.*

The distinct stages of dislocation structure development that undergoes during unloading of pre-strained M-oriented cluster contrast the stable defect arrangement disclosed for strained and unstrained C-GaN [Fig. S3 and Fig. 2(e)]. In a case of low pre-straining level (ε=0.127) the dislocations (green lines) introduced by compression [Fig. S5(a)] do not contract, but instead, develop further during unloading [Fig. S5(b-c)], to

* The plasticity of GaN reported so far was merely of local kind, *i.e*., certain mobile dislocations were observed [17], while the crystal as a whole remained rigid and brittle – similarly to the C-GaN case which we report.

finally annihilate themselves at the lateral surface of the frustum [Fig. S5(d)]. This results in defect-free structure of the unloaded M-GaN frustum, exactly as disclosed by our UHV-TEM observations [Fig. 2(e)].

The similar scenario occurs for the modestly (ε=0.129) and highly (ε=0.131) pre-strained M-GaN, where a number of dislocations is introduced during loading [Fig. S5(e) and (i)]. The defects () generated in the vicinity of the upper part of frustum, tend to develop during unloading and escape to its lateral surface, while part of these at the bottom entered the root substrate [see Fig. S5 (e)→(f)→(g)→(h) and (i)→(j)→(k)→(l)]. In the case of highly pre-strained M-GaN, our DXA-visualization captured a possibility of formation of an anchored dislocation. Such a defect may appear in an excessively strained M-GaN at intersection of different M-type slip planes and may reside in crystal even after complete unloading [Fig. S5(d)]. Generally however, the original, perfect, defect-free GaN structure strained along the M-direction to low- and moderate level, is restored during unloading, as we contend based on our UHV-TEM examinations and [Fig. 2(e)] and MD-simulations of stress-induced structural changes in GaN [Fig. S5(h) and (l)]. The presented results give a special credit to our DXA-analysis of MD-simulations capable to capture and explain nanoscale plasticity of GaN wurtzite nanoobjects.

D-3) *Plasticity of GaN nanocrystals in general context*

In summary, deformation of the strong, hard, wurtzite GaN ceramic/semiconductor nanocrystal usually develops along the 'classic path' that involves elastic straining followed by the limited plasticity stage, and finally cracking of the compressed nanopillar. Such a scenario is amply demonstrated by the C-GaN nanopillar when subjected to uniaxial compression along its C[0001] axis (Fig. 2c and 2e; Fig. 4a-c). The C-orientation of the crystal makes it impossible (at least at the initial stages of deformation) to activate either basal or prismatic slip. The process is highly dependent on dislocation nucleation, since an initially defect-free crystal exhibits a deficiency of linear defects. Once generated under increasing high stress, dislocations realize very limited, local pyramidal and rhombohedral slip (high Peierls barrier), being trapped inside the crystal. Finally, crack develops, relaxing the excessive stress, while the immobile dislocation remains within the structure.

In the case of M-GaN, the picture is entirely different, resembling to a certain extent the deformation of hexagonal metals. However, research on the Peierls stress reveals the disparity between dislocation-related properties of GaN and the hexagonal metals despite the apparent coincidence of the Wurtzite symmetry ($P6_3mc/6mm$) and hexagonal atom arrangement (Zn, Cd or Mg - *6mm*).

Moreover, the Peierls-Nabarro stress for the GaN prismatic M$\langle 11\bar{2}0\rangle\{1\bar{1}00\}$ slip, claimed by Kamimura *et al*. [53] as well as Yonenaga and Motoki [54] to be lower than the Peierls barrier for other slip systems, indicates the possibility of an easy glide on the M-planes. Thus, compression along the $\langle 1\bar{1}00\rangle$ direction of GaN enables the activation of the preferred slip, making dislocations quickly run out of the surface of the

pillar prior to new dislocations being nucleated. The process occurs exclusively in a nanoscale deconfined state (revealed by us for Si nanoparticles [7]) leading to dislocation starvation, similar to that observed for metals [19,20,46].

E. Supplementary references

S1. Godet, J., Pizzagalli, L., Brochard, S. & Beauchamp, P. Theoretical study of dislocation nucleation from simple surface defects in semiconductors. *Phys. Rev. B* **2004**, *70*, 054109.

S2. Tersoff, J. New empirical approach for the structure and energy of covalent systems. *Phys. Rev. B* **1988**, *37*, 6991 LP -7000.

S3. Godet, J., Pizzagalli, L., Brochard, S. & Beauchamp, P. Comparison between classical potentials and ab initio methods for silicon under large shear. *J. Phys. Cond. Matt.* **2003**, *15*, 6943-6953.

S4. Lu, H.; Meng, X. Correlation between band gap, electric constant, Young's modulus and melting temperature of GaN nanocrystals and their size and shape dependences. *Sci. Rep.* **2015**, *5*, 16939.

S5. D.L. Andrews, D.L.; Lipson, R.H.; Nann, T. *Comprehensive Nanoscience and Technology* (Academic Press 2019).

S6. Wagner, T.J.M.; Bechstedt, F. Properties of strained wurtzite GaN and AlN: *Ab initio* studies**.** *Phys. Rev. B* **2002**, *67*, 115202.

S7. Chen, M.; Wehrs, J.; Michler, J.; Wheeler, J.M. High-temperature *in situ* deformation of GaAs micro-pillars: Lithography versus FIB machining. *JOM* **2016**, *68*, 2761.

S8. Yokogawa, T.; Oya, M.; Fujikane, M. A high power InGaN-LED on an M-plane GaN substrate. *Electronics Comm. Jap*. **2015**, *98*, 1454.

S9. Fujikane, M.; Yokogawa, T.; Nagao, S.; Nowak, R. Strain rate controlled nanoindentation examination and incipient plasticity in bulk GaN crystal, *Jpn. J. Appl. Phys.* **2013**, *52*, 08JJ01.

S10. Fujikane, M.; Yokogawa, T.; Nagao, S.; Nowak, R. Yield shear stress dependence on nanoindentation strain rate in bulk GaN crystal, *Phys. Stat. Solidi* **2011**, *C8*, 429.

S11. Yokogawa, T.; Niki, S.; Maekawa, J.; Aoki, M.; Fujikane, M. Dislocation formation via an r-plane slip initiated by plastic deformation during nanoindentation of a high quality bulk GaN surface, *MRS Advances* **2016**, *1*, 3847.

S12. Yokogawa, T.; Fujikane, M.; Nagao, S.; Nowak, R. Mechanical and optical properties characterization of C-plane (0001) and M-plane (10−10) GaN by nanoindentation and luminescence. *MRS Proc.* **2015**, *1792*, Mrss15-2127895.

S13. Fujikane, M.; Leszczynski, M.; Nagao, S.; Nakayama, T.; Yamanaka, S.; Niihara, K.; Nowak, R. Elastic-plastic transition during nanoindentation in bulk GaN crystal, *J. Alloys & Comp.* **2008**, *450*, 405.

S14. Barnard, J.S.; Sharp, J.; Tong, J.R.; Midgley, P.A. Three-dimensional analysis of dislocation networks in GaN using weak-beam dark-field electron tomography. *Philos. Mag*. **2006**, *86*, 4901.

S15. E.B. Yakimov, E.B.; Vergeles, P.S.; Polyakov, A.; Lee, I-H.; Pearton, S.J. Radiation enhanced basal plane dislocation glide in GaN, *Jpn. J. Appl. Phys.* **2016**, *55*, 05FM03.

S16. Holt D.B.; Yacobi, B.G. *Extended Defects in Semiconductors: Electronic Properties, Device Effects and Structures*. Cambridge University Press (2007).

S17. Maeda, K.; Suzuki, K.; Ichihara, M.; Nishiguchi, S.; Ono, K.; Mera, Y.; Takeuchi, S. Electronically induced dislocation glide motion in hexagonal GaN single crystals. *Physica B* **1999**, *273-274*, 134.

S18. Nord, J.; Albe, K.; Erhart, P.; Nordlund, K. Modelling of compound semiconductors: Analytical bond-order potential for gallium, nitrogen and gallium nitride. *J. Phys. Condens. Matter*. **2003**, *15*, 5649.

F. Supplementary Figures

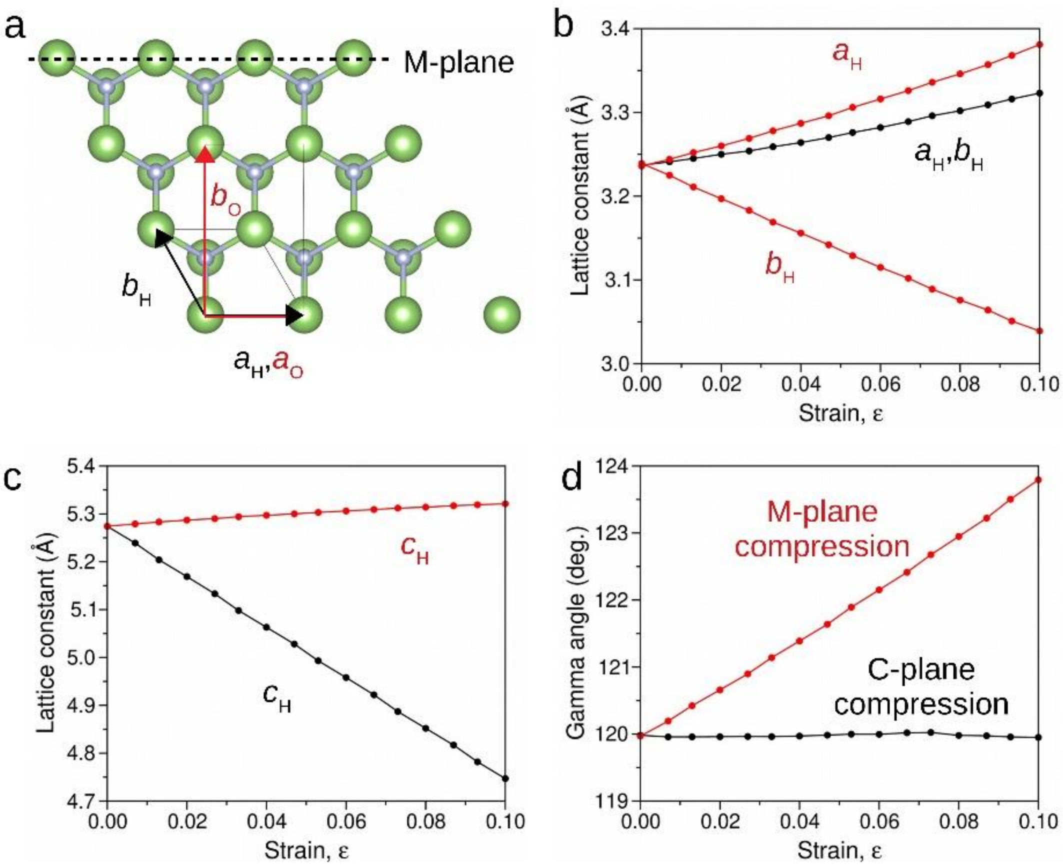

Fig. S1.

Strain-dependent changes of GaN lattice parameters deduced from our *ab initio* calculations.

(**a**) The modelling of GaN compression along the C[0001]-axis was accomplished with hexagonal wurtzite structure (characterized by unit cell vectors $\boldsymbol{a_H}$, $\boldsymbol{b_H}$ marked in black), while the squeezing along the M[$10\bar{1}0$] direction demanded orthogonal lattice (defined by the $\boldsymbol{a_0}$ and $\boldsymbol{b_0}$vectors marked in red). The third unit cell vector ($\boldsymbol{c_H} = \boldsymbol{c_0}$) was in each case perpendicular to both $\boldsymbol{a}$ and $\boldsymbol{b}$ directions. Thus, the compression in C-direction involved the stimulated changes of $\boldsymbol{c_H}$, and the subsequent adjustment (relaxation) of $\boldsymbol{a_H}$ and $\boldsymbol{b_H}$ parameters associated with a location of atoms in stressed structure. In contrast, variations of $\boldsymbol{b_0}$ lattice constant followed by concerted relaxation of $\boldsymbol{a_0}$ and $\boldsymbol{c_0}$ enabled us realize the compression of GaN orthogonal unit cell along the M-direction. The variations of $\boldsymbol{b_H}$, $\boldsymbol{c_H}$, and the angle γ between $\boldsymbol{a_H}$and $\boldsymbol{b_H}$ vectors for uniaxially stressed GaN structure are shown in graphs denoted as (**b**) (**c**) and (**d**) graphs. In the case of the compression along the M-direction, the relaxed vectors of orthogonal unit cell were expressed in hexagonal coordination.

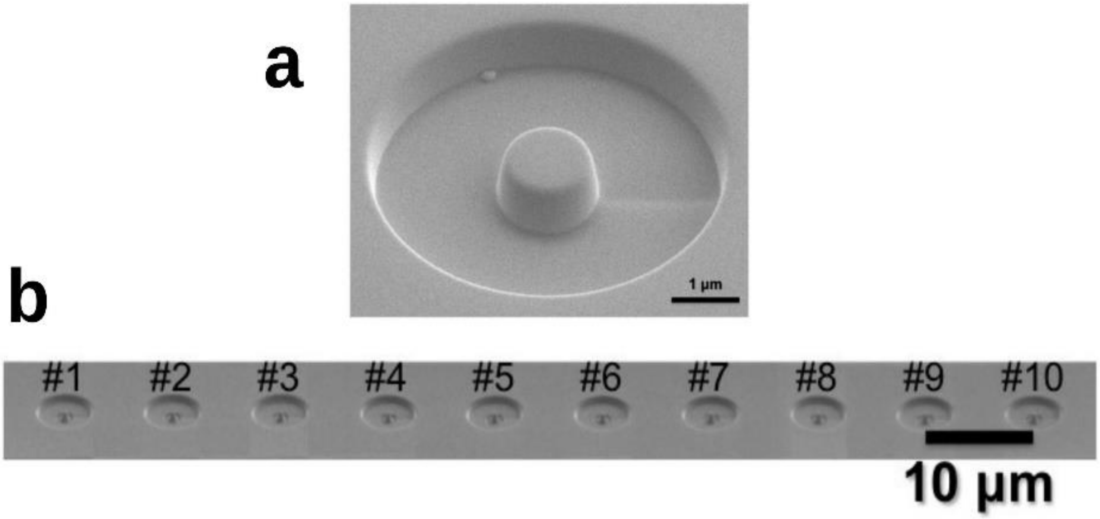

Fig. S2.

Two-stages process of GaN nanopillars fabrication using FIB technique.

(**a**) The surface of GaN after the first stage of FIB-cutting. The wide crater enables us to use a large radius spherical tip of the nanoindenter to accomplish compression. The presented SEM micrograph proves the perfect nature of the crystal surface used to fabricate GaN nanoobjects. (**b**) A general SEM-view of the set of ten identical M-GaN nanopillars fabricated by FIB-milling. The maintained distance among pillars insures unaffected deformation.

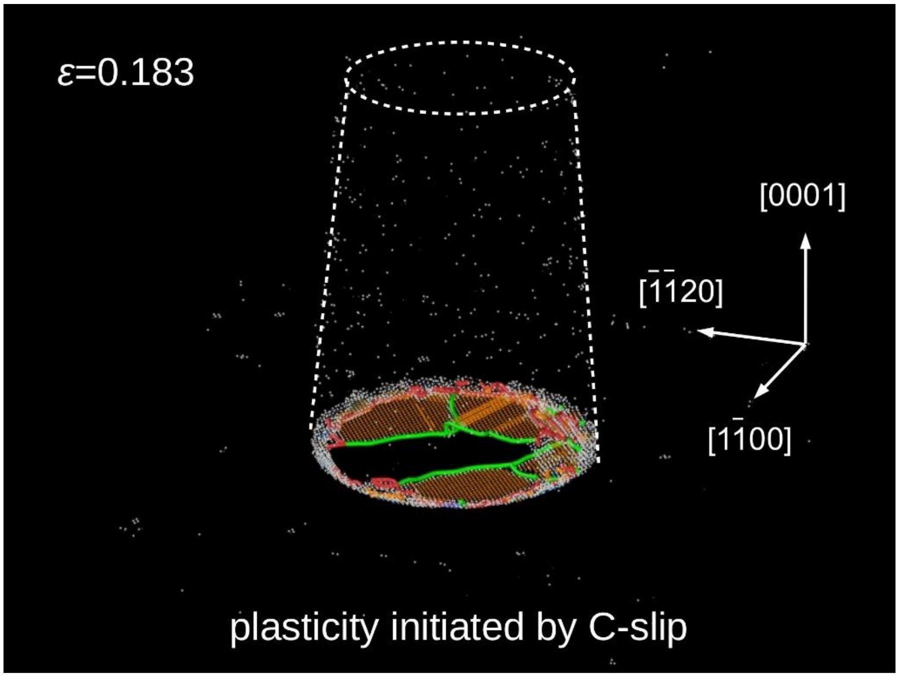

Fig. S3.

DXA image of dislocations nucleated in the C-GaN nanopillar at the onset of plastic deformation.

Applying the DXA visualization method by Stukowski [51] to analyze the results of our MD-simulations, led to the detection of the initial dislocations (green lines) generated in the C-oriented, frustum GaN cluster which signify its incipient plasticity [see Fig. 3 and Fig. 4(a)]. The brown-colored atoms show the C(0001) plane as the preferred slip plane at the beginning of the C-GaN's irreversible deformation.

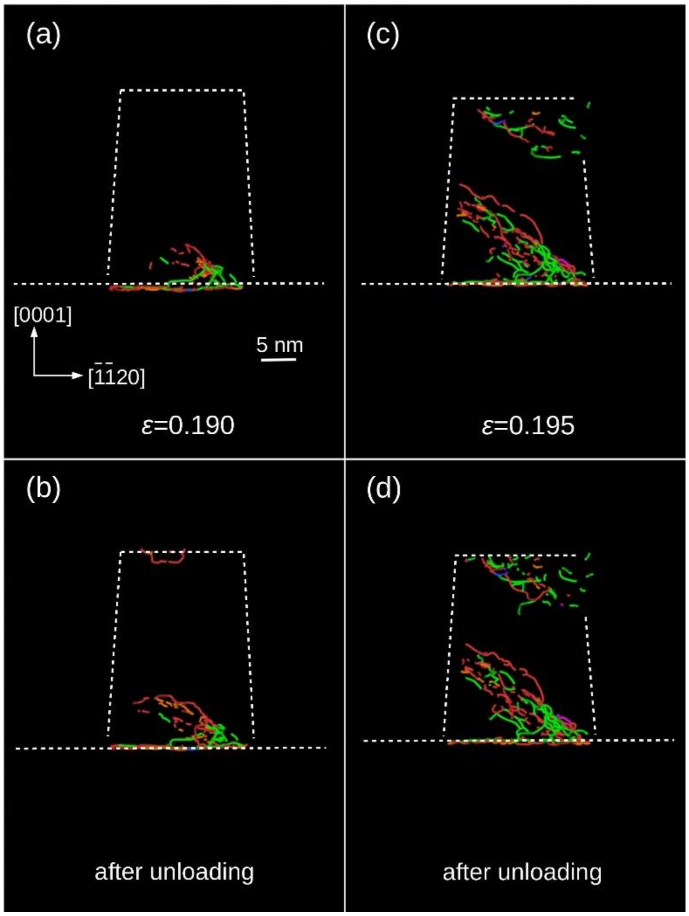

Fig. S4.

DXA images of the evolution of dislocations in the C-oriented GaN during unloading.

The frustum C-GaN cluster originally strained up to ε = 0.190 (a) and ε = 0.195 (b) conserves its initial dislocation structure after unloading (b)(d). The defects remain arrested in the C-oriented GaN crystal - exactly as borne out by UHV-TEM observations [Fig. 2(e)]. Interestingly, the dislocations generated during the loading cycle (a)(b) do not enter into the root-substrate - again in accord with our experiment [Fig. 2(e)]. We determined the Burgers vectors *b* of the ‘green dislocation lines’ as b=1/3$\langle 11\bar{2}0\rangle$, while those associated with the ‘red dislocation lines’ as either b=1/3$\langle 11\bar{2}3\rangle$ or b=1/6$\langle 20\bar{2}3\rangle$.

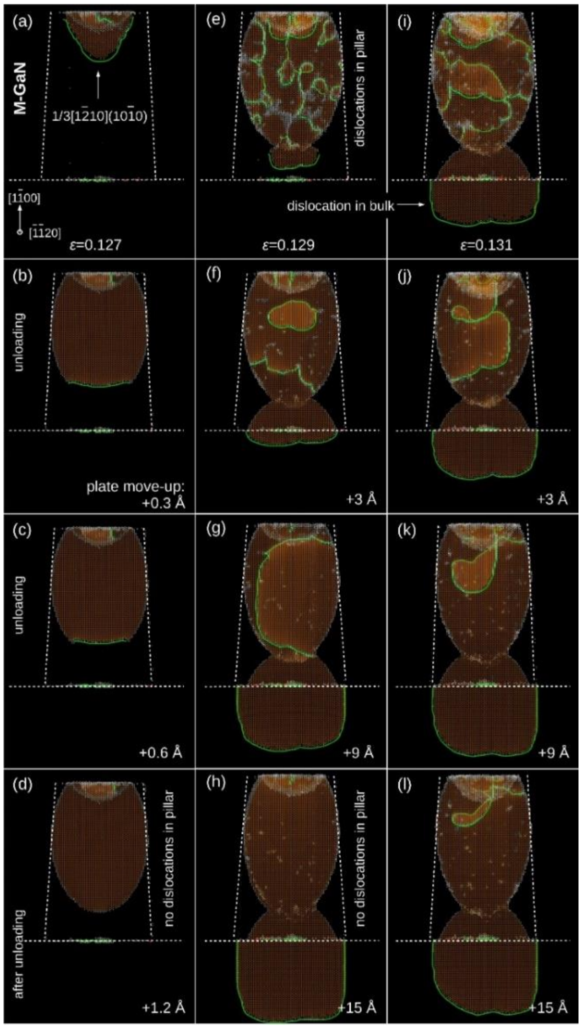

Fig. S5.
DXA images of the evolution of the dislocation structure in the variously strained, M-oriented GaN cluster during unloading.

The frustum M-GaN clusters strained up to ε = 0.127 (a), ε = 0.129 (e) and ε = 0.131 (i) relax during unloading realized by gradual lifting of the horizontal squeezing plane*. A visualization of the dislocation structure is provided for a sequence of tool positions during loading: 0.0→0.3→0.6→1.2 Å for the M-pillar pre-strained up to ε = 0.127 (a-d) and during unloading: 0.0→3.0→9.0→1.2 Å for the M-GaN strained up to ε = 0.129 (e-h) and ε = 0.131 (i-l). Our visualization demonstrates that compression-induced dislocations escape during the M-GaN unloading either to the pillar's lateral surface or else enter the root-substrate. This leaves behind a virtually defect-free pillar volume (d, h), as revealed by our UHV-TEM examination (Fig. 2), with a single, sessile dislocation in an excessively pre-strained cluster (l). An extended version of the presented images is displayed in the main text as Fig. 5. All dislocations concern the M-plane slip of defects with the Burgers vector b=1/3$\langle 11\bar{2}0\rangle$.

* The horizontal, rigid plane that induces compression of the GaN frustum is not displayed in the figure.

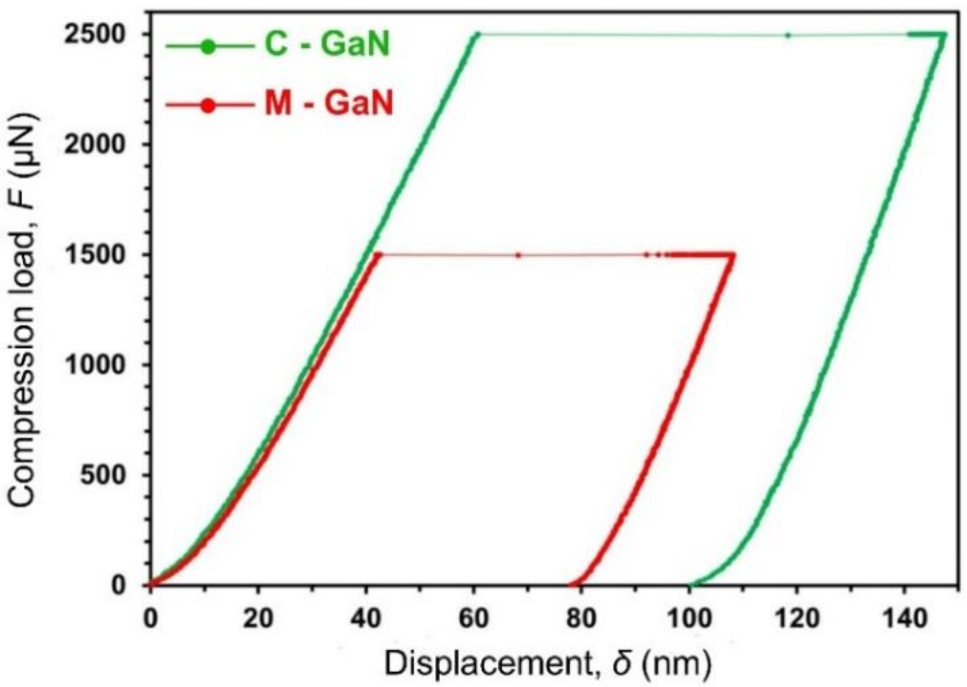

Fig. S6.

The nanocompression load-displacement (*F*-δ) for the C-GaN and M-GaN nanopillars (*raw data*).

With the help of recorded load-displacement (*F*-δ) data, the stress-strain (σ–ε) relationship for a single-pop-in behaviour was determined using the procedure developed for compressed Si nanospheres [7].

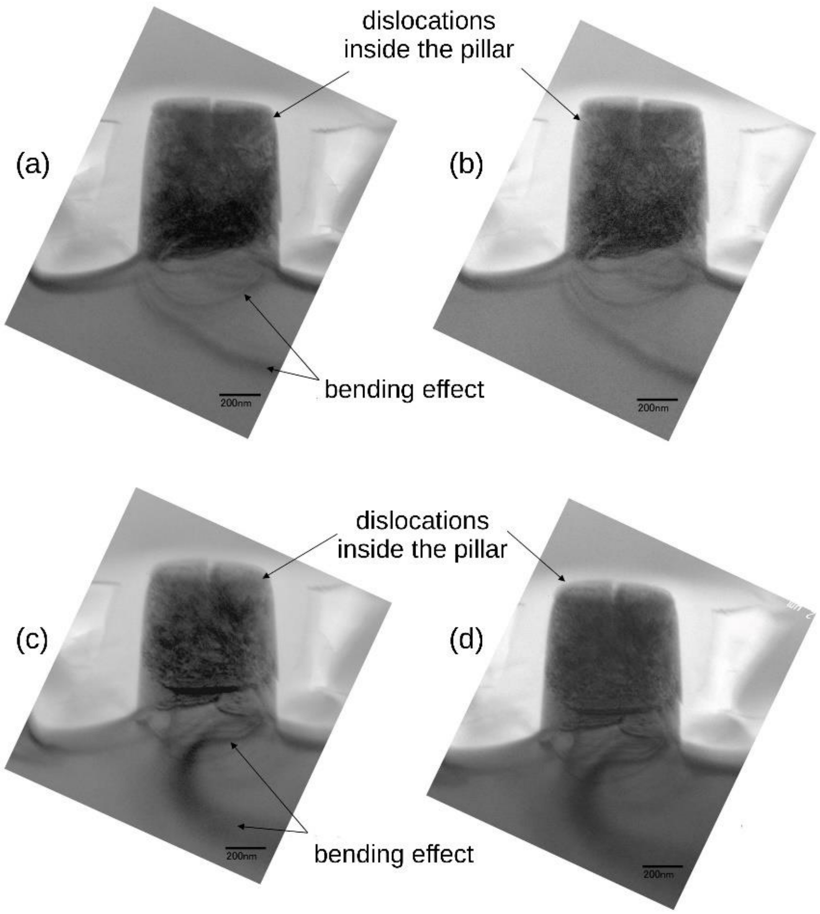

Fig. S7.

The bright field UHV-TEM views of C-GaN pillar structure after a single strain-burst deformation. The micrographs demonstrate dislocation structure of the nanopillar at inclinations a=10º (a) (b) and a=0º (c)(d). In addition to conventional transmission observations (a)(c), the pillar was examined (b)(d) using energy filtered technique (EF-TEM, ΔE = 60 eV).

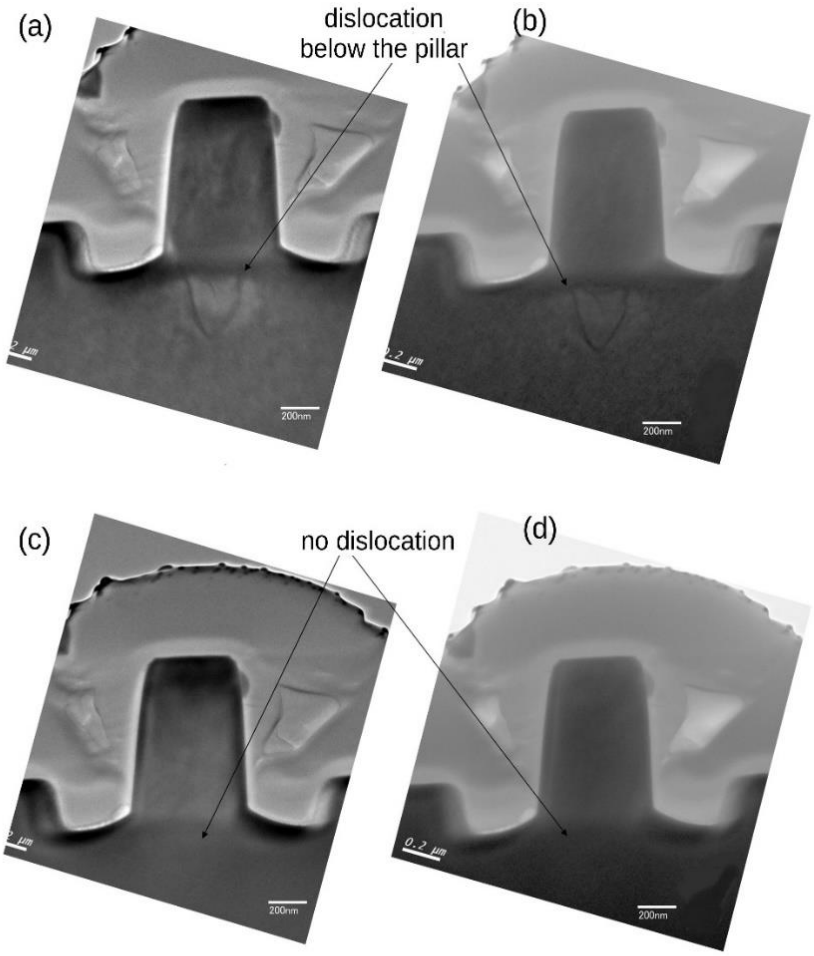

Fig. S8 .

The bright field UHV-TEM views of M-GaN pillar structure after a single strain-burst deformation. The micrographs reveals structure of the nanopillar at inclinations a=10° (a) (b) and a=0° (c)(d). In addition to conventional transmission observations (a)(c), the pillar was examined (b)(d) using energy filtered technique (EF-TEM, ΔE = 60 eV). The dislocations are not detected in the volume of M-GaN nanoobjects.
